# Tolerance response of multidrug-resistant *Salmonella enterica* strains to habituation to *Origanum vulgare* L. essential oil

**DOI:** 10.3389/fmicb.2014.00721

**Published:** 2014-12-19

**Authors:** Daniel F. M. Monte, Adassa G. Tavares, Allan R. Albuquerque, Fábio C. Sampaio, Tereza C. R. M. Oliveira, Octavio L. Franco, Evandro L. Souza, Marciane Magnani

**Affiliations:** ^1^Department of Food Engineering, Center of Technology, Federal University of ParaibaJoão Pessoa, Brazil; ^2^Department of Nutrition, Center of Health Sciences, Federal University of ParaibaJoão Pessoa, Brazil; ^3^Department of Clinical and Social Dentistry, Center of Health Sciences, Federal University of ParaibaJoão Pessoa, Brazil; ^4^Department of Food Science and Technology, Center of Agricultural Sciences, Londrina State UniversityLondrina, Brazil; ^5^Center of Biochemical and Proteomic Analysis, Catholic University of BrasíliaBrasília, Brazil; ^6^S-Inova, Pós-Graduação em Biotecnologia, Universidade Católica Dom BoscoCampo Grande, Brazil; ^7^Laboratory of Microbial Processes in Foods, Department of Food Engineering, Center of Technology, Federal University of ParaibaJoão Pessoa, Brazil

**Keywords:** *Salmonella*, oregano, tolerance, ciprofloxacin, multidrug-resistant

## Abstract

Multidrug-resistant *Salmonella enterica* isolates from human outbreaks or from poultry origin were investigated for their ability to develop direct-tolerance or cross-tolerance to sodium chloride, potassium chloride, lactic acid, acetic acid, and ciprofloxacin after habituation in subinhibitory amounts ( of the minimum inhibitory concentration – (MIC) and of the minimum inhibitory concentration – MIC) of *Origanum vulgare* L. essential oil (OVEO) at different time intervals. The habituation of *S. enterica* to OVEO did not induce direct-tolerance or cross-tolerance in the tested strains, as assessed by the modulation of MIC values. However, cells habituated to OVEO maintained or increased susceptibility to the tested antimicrobials agents, with up to fourfold double dilution decrease from previously determined MIC values. This study reports for the first time the non-inductive effect of OVEO on the acquisition of direct-tolerance or cross-tolerance in multidrug-resistant *S. enterica* strains to antimicrobial agents that are largely used in food preservation, as well as to CIP, the therapeutic drug of salmonellosis.

## INTRODUCTION

*Salmonella enterica* is recognized as the most frequent cause of foodborne disease in the world (Hendriksen et al., 2011; Gomes-Neto et al., 2014). Foodborne outbreaks caused by this pathogen are mainly associated with the consumption of chicken meat, eggs, and egg products (Kottwitz et al., 2010; Timme et al., 2012). A number of studies have shown that previous exposure of *S. enterica* to a single stressful condition could result in homologous or heterologous tolerance and increasing subsequent resistance to the same or different environment stresses (Álvarez-Ordóñez et al., 2010; Dubois-Brissonnet et al., 2011; Shah et al., 2013). Salts and acids are traditionally used to process and preserve food, and these compounds impose stress conditions on bacterial cells to limit their growth and survival. However, it has been reported that the exposure of *S. enterica* to subinhibitory conditions imposed by these classical antimicrobial compounds can undergo genetic and physiologic changes. These changes allow the cells to become more resistant in subsequent exposures to the antimicrobial compounds, due to the development of a tolerance response (Álvarez-Ordóñez et al., 2012; Spector and Kenyon, 2012; Yang et al., 2014).

One of the concerns related to the increase of antimicrobial resistance in *Salmonella* is the reduction of the clinical efficacy of antibiotics, particularly the quinolones, resulting in treatment failure. Ciprofloxacin (CIP) is a 2^nd^ generation quinolone, is one of the first-choice agents used to treat salmonellosis. However, a significant rise in the number of *Salmonella* strains with reduced susceptibility to this antibiotic has been observed in humans and food involved outbreaks (Ferrari et al., 2013a; Rushdy et al., 2013; Ballesté-Delpierre et al., 2014).

*Origanum vulgare* L. essential oil (OVEO) has been cited as a potential compound to control the growth and survival of *S. enterica* in food (Gomes-Neto et al., 2014; Luz et al., 2014), due to strong anti-*Salmonella* activity, as shown in laboratory media and in food-mimicking models (Álvarez-Ordóñez et al., 2009; Luz et al., 2012). However, there is a lack of information regarding the effects of exposure to OVEO on the development of tolerance in multidrug-resistant *S. enterica* to antimicrobials traditionally used to prevent the infection of this bacterium in food industry or for therapeutic treatment of salmonellosis. Knowledge about the magnitude of tolerance induction in foodborne pathogens, particularly in *S. enterica*, which possesses a large diversity of resistance mechanisms (Spector and Kenyon, 2012), must be a requisite for the development of anti-bacterial compounds, such as OVEO, that are considered for application in food preservation systems.

Considering these aspects, the aim of this study was to assess the effects of the exposure of multidrug-resistant epidemic *S. enterica* strains to subinhibitory concentration of OVEO for different time intervals on the development of bacterial tolerance to salts and organic acids used by the food industry, as well to CIP, a therapeutic drug of human salmonellosis.

## MATERIALS AND METHODS

### ESSENTIAL OIL AND ANTIMICROBIALS AGENTS

The antimicrobial agents used in this study were OVEO (Laszlo Aromaterapia Ltda., Minas Gerais, Brazil), CIP (Oxoid, UK), sodium chloride (NaCl P.A.), potassium chloride (KCl), glacial acetic acid (AA), and lactic acid (LA, 85%; Vetec Ltda., Rio de Janeiro, Brazil). According to the technical report presented by the supplier, carvacrol is the most prevalent compound in the OVEO assayed in this study (66.1 g/100 mL), followed for *p*-cymene (12.4 g/100 g) and γ-terpinene (8.3 g/100 g). OVEO solutions (40–0.3 μL mL^-1^) were prepared in sterile brain heart infusion (BHI) broth (Himedia, India) and Tween 80 (1%; Sigma–Aldrich, USA) was added as an emulsifier. Preliminary test were conducted to ensure that the antibacterial activity was because of the OVEO and not Tween 80. The results demonstrated that Tween 80 at the given concentration did not inhibit the growth of the assayed bacterial strains cultivated in BHI broth. Solutions of NaCl (600–50 mg mL^-1^), KCl (600–50 mg mL^-1^), AA (160–1.25 μL mL^-1^), and LA (160–1.25 μL mL^-1^) were prepared in sterile BHI broth.

### TEST STRAIN

The test microorganisms used in this study included *S*. Enteritidis 209, isolated from feces of outbreak patient, *S.* Typhimurium 149, isolated from food involved in outbreak and *S.* Corvallis 297 isolated from poultry (chicken hens). The strains were resistant to aminoglycosides (gentamicin and streptomycin); β-lactams (ampicillin and cefotaxime); quinolones (norfloxacin and nalidixic acid), and sulphametoxazole-trimethoprim. The strains were characterized according to their quinolone resistance mechanism(s) as described in **Table 1** (Souza et al., 2011; Ferrari et al., 2013a,b). Stock cultures were kept at 4 C before use. Each strain was grown in BHI broth at 37°C for 18 h (late exponential growth phase), harvested by centrifugation (4500 *g,* 15 min, 4°C), washed twice in sterile saline solution (NaCl, 0.85%), and resuspended in sterile saline solution to obtain standard cell suspensions at which the OD reading at 660 nm (OD_660_) was 0.1 (c.a. 10^7^ CFU mL^-1^; McMahon et al., 2008).

**Table 1:** Resistance mechanisms and the minimum inhibitory concentration (MIC) of essential oil from *Origanum vulgare* L. and ciprofloxacin (CIP) against multidrug-resistant *Salmonella enterica* strains isolated from human outbreaks or from poultry origin.

Test strains	Resistance mechanism*	MIC** of OVEO (μL mL^-1^)	MIC* of CIP (μL mL^-1^)
*S.* Enteritidis 209	Ser 83-Tyr *gyrA* gene mutation	2.5	0.12
	Active AcrAB-TolC system	0	0
*S.* Typhimurium 149	Ser 83-Tyr *gyrA* gene mutation	2.5	0.12
*S.* Corvallis 297	PMRQ- *qnr*B1 plasmidial gene	5	0.5

*Resistance Mechanism previously characterized by Souza et al. (2011) and Ferrari et al. (2013a,b); **MIC: Minimum Inhibitory Concentration; OVEO, *O. vulgare* L. essential oil; CIP, cirpofloxacin.

### DETERMINATION OF MINIMUM INHIBITORY CONCENTRATION (MIC)

A modified microtiter plate assay was used to determine the MIC of OVEO, NaCl, KCl, AA, LA, and CIP (Sarker et al., 2007). The 96-well plates were prepared by dispensing 90 μL of OVEO (40–0.31 μL mL^-1^), salts (600–50 mg mL^-1^), acids (16–0.125 μL mL^-1^), or CIP solutions (0.005 μL mL^-1^ to 1 μL mL^-1^) into 90 μL of double-concentration BHI broth in each well. Finally, 10 μL of bacterial suspension (10^7^ CFU mL^-1^) were added to each well. The microplate was wrapped loosely with cling wrap to prevent bacterial dehydration and ensure that OVEO would not volatilize. Each plate included controls without the antimicrobial test agents. The plates were prepared in triplicate, and they were statically incubated at 37°C for 24 h in a microplate incubator/reader (EON model, Biotek Inc., USA). Following incubation, MIC values were confirmed as the lowest concentrations of OVEO, NaCl, KCl, AA, LA, or CIP at which the OD_660_ was less than 0.01 (McMahon et al., 2008). Breakpoints that were designed by Clinical and Laboratory Standards Institute (CLSI), (2012) were used to interpret MIC values of CIP [Clinical and Laboratory Standards Institute (CLSI), 2012]. All MIC determination assays were performed in triplicate and in three separate experiments. The results were expressed as modal values because no variation was observed between the replicated results (McMahon et al., 2007).

### EVALUATION OF INDUCTION OF BACTERIAL DIRECT-TOLERANCE AND BACTERIAL CROSS-TOLERANCE

The induction of direct-tolerance and cross-tolerance was performed by exposing the test strains to subinhibitory OVEO concentrations for different time intervals, followed by a determination of the MIC values for the homologous stressing agent (OVEO) or heterologous stressing agents (NaCl, KCl, AA, LA, and CIP; Luz et al., 2012). For this test, 4 mL of BHI broth was inoculated with 1 mL of bacterial suspension (c.a. 10^7^ CFU mL^-1^). Appropriate amounts of OVEO was added to obtain the desired final concentration ( MIC or MIC), followed by static incubation at 37°C. After 24, 48, and 72 h of incubation, an aliquot of each system was taken (standardized to OD_600_ values of 0.1, c.a. 10^7^ CFU mL^-1^ of habituated cells) as inoculum (10 μL) to determine the MIC of OVEO, or MIC of NaCl, KCl, AA, and LA using the same microtiter plate assay as described before (Sarker et al., 2007). The induction of direct-tolerance and cross-tolerance in bacteria was tested by comparing the MIC values of OVEO or NaCl, KCl, AA, LA, and CIP against those of the tested strains before and after the habituation treatment with subinhibitory amounts of OVEO. Control systems without OVEO exposure were similarly tested (non-habituation treatment). All assays were performed in triplicate in three separate experiments, and the results were expressed as modal or median values. Only the modal values were presented in those experiments yielding same results (McMahon et al., 2007). Significant differences (*P* < 0.05) for induction of tolerance were considered when changes in MIC values were equal to or higher than a twofold increase (≥twofold increase; Hammer et al., 2012).

## RESULTS

The MIC values of OVEO against the tested strains ranged from 2.5 μL mL^-1^ to 5 μL mL^-1^. Lower susceptibility to OVEO was observed in *S.* Corvallis 297 compared to *S.* Enteritidis 209 and *S.* Typhimurium 149 (**Table 1**). MIC of CIP ranged from 0.12 μL mL^-1^ to 0.5 μL mL^-1^, which is considered to be a phenotype of reduced susceptibility to CIP according to the breakpoints designed by Clinical and Laboratory Standards Institute (CLSI), (2012). Similar to OVEO, the highest MIC of CIP was also observed against *S.* Corvallis 297. NaCl, KCl, AA, and LA yielded MIC values of 150 mg mL^-1^, 200 mg mL^-1^, 2.5 μL mL^-1^, and 10 μL mL^-1^, respectively, against all the assayed strains.

The habituation of the strains to OVEO during the assessed time-intervals (24, 48, and 72 h) caused a decrease in MIC values of all strains studied. Regardless of the tested *Salmonella* strain and the OVEO concentration used for habituation ( MIC or MIC; **Table 2**), there was no induction of direct-tolerance in the bacterial cells for 72 h. The maximum decrease in MIC value of OVEO (fourfold double dilution) was observed in *S*. Corvallis (5 μL mL^-1^ to 0.3 μL mL^-1^; **Table 2**). The decreased MIC values of OVEO against habituated *S. enterica* was related to the time of exposure to subinhibitory concentrations of OVEO because, with exception of *S.* Typhimurium 149 habituated to MIC of OVEO, the smallest MIC values were generally found against cells that were pre-exposed to OVEO for 72 h when compared with non-habituated. During all the assessed time intervals, the MIC values of OVEO against non-habituated cells ranged from 5 μL mL^-1^ to 2.5 μL mL^-1^.

**Table 2:** The MIC of the essential oil from *O. vulgare* L. against multidrug-resistant *S. enterica* strains isolated from human outbreaks or from poultry origin, with or without habituation to the same stressing agent for 72 h.

Strains	Treatment	MIC (μL mL^-1^)		
		24 h*	48 h*	72 h*
*S.* Enteritidis 209	Control (0 μL OVEO mL^-1^)	2.5	2.5	2.5
	MIC OVEO (1.25 μL OVEO mL^-1^)	2.5	1.25	0.3
	MIC OVEO (0.6 μL OVEO mL^-1^)	2.5	1.25	0.3
*S.* Typhimurium 149	Control (0 μL OVEO mL^-1^)	2.5	2.5	2.5
	MIC OVEO (1.25 μL OVEO mL^-1^)	1.25	1.25	0.6
	MIC OVEO (0.6 μL OVEO mL^-1^)	2.5	2.5	2.5
*S.* Corvallis 297	Control (0 μL OVEO mL^-1^)	5	5	5
	MIC OVEO (2.5 μL OVEO mL^-1^)	2.5	1.25	0.3
	MIC OVEO (1.25 μL OVEO mL^-1^)	2.5	2.5	2.5

*Hours of previous habituation or not in the assayed sublethal concentrations of *O. vulgare* L. essential oil; MIC, minimum inhibitory concentration; OVEO, *O. vulgare* L. essential oil.

Similar to the direct-tolerance results, MIC values for NaCl, KCl, AA, and LA against the OVEO-habituated cells were the same or decreased (one to threefold double dilution), when compared with MIC values against non-habituated cells (**Tables 2 and 3**). There was no effect of time-of-habituation to OVEO on the sensitivity of the habituated cells to NaCl, KCl, and LA. However, the decrease in MIC values of AA against habituated-cells always occurred after 72 h of exposure to subinhibitory concentration of OVEO. MIC values of CIP against the habituated cells were maintained when compared with the previously determined MIC values, indicating that habituation to OVEO did not alter the sensitivity of the strains to CIP. A transient decrease (two-fold double dilution) of MIC values of CIP against *S.* Enteritidis 209 was observed after 48 h of habituation to MIC and MIC of OVEO.

**Table 3:** Minimum inhibitory concentrations of sodium chloride, potassium chloride, acetic acid, and lactic acid against multidrug-resistant *S. enterica* strains isolated from human outbreaks or from poultry origin, with or without habituation to the essential oil from *O. vulgare* L. for 72 h.

Strains	Treatment	Sodium chloride (NaCl) MIC (mg mL^-1^)			Potassium chloride (KCL) MIC (mg mL^-1^)			Acetic acid MIC (μL mL^-1^)			Lactic acid MIC (μL mL^-1^)			Ciprofloxacin (CIP) MIC (μL mL^-1^)		
		24 h*	48 h*	72 h*	24 h*	48 h*	72 h*	24 h*	48 h*	72 h*	24 h*	24 h*	24 h*	24 h*	48 h*	72 h*
*S.* Enteritidis 209	Control (0 μL OVEO mL^-1^)	150	150	150	200	200	200	2.5	2.5	2.5	10	10	10	0.12	0.12	0.12
	MIC OVEO (1.25 μL OVEO mL^-1^)	150	75	75	200	100	75	2.5	2.5	1.25	5	5	1.25	0.12	0.5	0.12
	MIC OVEO (0.6 μL OVEO mL^-1^)	150	75	75	200	200	75	2.5	2.5	1.25	5	5	1.25	0.12	0.5	0.12
																
*S.* Typhimurium 149	Control (0 μL OVEO mL^-1^)	150	150	100	200	200	200	2.5	2.5	2.5	10	5	5	0.12	0.12	0.12
	MIC OVEO (1.25 μL OVEO mL^-1^)	150	100	75	100	75	50	2.5	2.5	2.5	5	5	5	0.12	0.12	0.12
	MIC OVEO (0.6 μL OVEO mL^-1^)	150	150	150	200	200	100	2.5	2.5	2.5	5	5	5	0.12	0.12	0.12
																
*S.* Corvallis 297	Control (0 μL OVEO mL^-1^)	150	150	100	200	200	200	2.5	2.5	2.5	10	5	5	0.5	0.5	0.5
	MIC OVEO (2.5 μL OVEO mL^-1^)	50	50	50	200	200	50	2.5	2.5	1.25	5	5	1.25	0.5	0.5	0.5
	MIC OVEO (1.25 μL OVEO mL^-1^)	50	50	50	200	200	50	2.5	2.5	1.25	5	5	1.25	0.5	0.5	0.5

*Hours of previous habituation (or not) to *O. vulgare* L. essential oil at the assayed sublethal concentrations; MIC, minimum inhibitory concentration; OVEO, *O. vulgare* L. essential oil.

## DISCUSSION

*Salmonella enterica* presents a dynamic interaction with host and environment and has a high genetic variability related to the development of different antimicrobial resistance mechanisms (Ferrari et al., 2013a; Du et al., 2014). The serovars *S.* Enteritidis and *S.* Typhimurium are frequently cited as more tolerant to many antimicrobials agents when compared to other *Salmonella* serovars (Souza et al., 2011; Ferrari et al., 2013b; Ballesté-Delpierre et al., 2014). However, in the present study MIC values of OVEO and CIP were higher against *S.* Corvallis 297 when compared to the values against *S.* Enteritidis 209 and *S.* Typhimurium 149. The presence of *qnr*B19 gene in *S.* Corvalis 297 (Ferrari et al., 2013a), a well-known mechanism of resistance to quinolone in *Salmonella*, could be related to the higher tolerance of the strains to the substances that were tested.

*Salmonella* Corvallis 297 showed the most decrease (fourfold double dilution) in MIC values of OVEO after habituation for 72 h and was the only strain that exhibited a significant decrease [more than onefold double dilution; Clinical and Laboratory Standards Institute (CLSI), 2012] in MIC of NaCl and this decrease occurred at the 24 h time point (**Tables 1 and 2**). The behavior of *S.* Corvallis could be associated with the low expression of AcrAB-TolC eﬄux pump related genes that has been previously described in this strain (Ferrari et al., 2013b). AcrAB-TolC is a well-known eﬄux system in *S. enterica* which is able to help in extrusion of bile salts, lipophilic antibiotics, dyes, detergents, and solvents from the cell. The expression of genes related to this eﬄux system has been associated with multiple-drug resistance (Spector and Kenyon, 2012), which is observed among the *Salmonella* strains assayed in this study. However, this explanation is in contrast with the behavior of *S.* Enteritidis 209, in which, despite high expression levels of AcrAB-TolC eﬄux pump related genes, the sensitivity to OVEO was increased (threefold double dilution decrease in MIC value) but the sensitivity to NaCl remained unaltered after habituation (Ferrari et al., 2013b). Changes in the cell wall structure and increased membrane permeability have been observed in Gram-negative bacteria that were exposed to sublethal concentrations of OVEO (Souza et al., 2013). These alterations in membrane structure, caused by the exposure to OVEO, could have affected the osmoregulation ability of the membrane or its capacity to extrude toxic materials and, therefore, increased the sensitivity of the tested *Salmonella* strains to OVEO, salts and acids. Changes in the cellular membrane fatty acids composition of *S.* Typhimurium that were subjected to habituation to OVEO at subinhibitory concentrations have been reported earlier (Luz et al., 2014). Even if an adaptation-response related changes in membrane occurred in the tested strains, these changes were not capable of causing obvious increase (≥twofold doubling dilution; Hammer et al., 2012) in MIC values of OVEO, salts and acids, and therefore, conferring the development of direct-tolerance or cross-tolerance. In this study, non-induction of cross-tolerance to salts and acids after habituation to OVEO is noteworthy because the development of homologous and heterologous tolerance in *S. enterica* that were challenged with subinhibitory conditions, provided by other antimicrobial compounds or procedures used for controlling microbial growth and survival in foods, has already been documented (Dubois-Brissonnet et al., 2011). Previous studies have found development of cross-tolerance in *S. enterica* after habituation to LA (Leyer and Johnson, 1993), AA (Álvarez-Ordóñez et al., 2009, 2012), NaCl, and KCl (Greenacre and Brocklehurst, 2006).

Maintenance of susceptibility to CIP after exposure to OVEO for an extended period was noticeable in the tested *Salmonella* strains. The multidrug-resistant phenotypes of these strains, with resistance to a variety of antibiotic classes that target different cellular components including the cell membrane, make this observation distinctive. Changes in antibiotic susceptibility in *S.* Typhimurium ST11 and *S.* Enteritidis NCTC 12694 after habituation to subinhibitory concentrations of *Melaleuca alternifolia* L. essential oil have been reported earlier. These changes in antibiotic susceptibility were associated with increase (≥twofold double dilution) in MIC values of gentamicin, erythromycin, chloramphenicol, tetracycline, streptomycin, and trimethoprim (McMahon et al., 2008).

To our knowledge, this is the first study that investigated the tolerance development by multidrug-resistant *S. enterica* strains after habituation to subinhibitory concentrations of OVEO. These results revealed that habituating *S. enterica* strains to subinhibitory amounts of OVEO maintained or increased susceptibility to the same stressing agent and also to the tested heterologous stressing agents (NaCl, KCl, LA, AA, and CIP).

## Conflict of Interest Statement

The authors declare that the research was conducted in the absence of any commercial or financial relationships that could be construed as a potential conflict of interest.

## References

[B1] Álvarez-Ordóñez A. Fernández A. Bernardo A. López M. (2009). A comparative study of thermal and acid inactivation kinetics in fruit juices of *Salmonella enterica* serovar Typhimurium and *Salmonella enterica* serovar Senftenberg grown at acidic conditions. *Foodborne Pathog. Dis.* 6 1147–1155 10.1089/fpd.2009.0313 19694554

[B2] Álvarez-Ordóñez A. Fernández A. Bernardo A. López M. (2010). Arginine and lysine decarboxylases and the acid tolerance response of *Salmonella* Typhimurium. *Int. J. Food Microbiol.* 136 278–282 10.1016/j.ijfoodmicro.2009.09.024 19864032

[B3] Álvarez-Ordóñez A. Prieto M. Bernardo A. Hill C. López M. (2012). The acid tolerance response of *Salmonella* spp: an adaptive strategy to survive in stressful environments prevailing in foods and the host. *Food Res. Int.* 45 482–492 10.1016/j.foodres.2011.04.002

[B4] Ballesté-Delpierre C. Solé M. Domènech O. Borrell J. Vila J. Fàbrega A. (2014). Molecular study of quinolone resistance mechanisms and clonal relationship of *Salmonella enterica* clinical isolates. *Int. J. Antimicrobial Agents* 43 121–125 10.1016/j.ijantimicag.2013.08.017 24139882

[B5] Clinical and Laboratory Standards Institute (CLSI). (2012). “Performance standard for antimicrobial susceptibility testing,” in *Twenty-Second Information Supplement* Vol. 32. Wayne: CLSI/NCCLS.

[B6] Du D. Wang Z. James N. R. Voss J. E. Klimontt E. Ohene-Agyei T. (2014). Structure of the AcrAB–TolC multidrug eﬄux pump. *Nature* 509 512–515 10.1038/nature13205 24747401 PMC4361902

[B7] Dubois-Brissonnet F. Naïtali M. Mafu A. A. Briandet R. (2011). Induction of fatty acid composition modifications and tolerance to biocides in *Salmonella enterica* serovar Typhimurium by plant-derived terpenes. *Appl. Environ. Microbiol.* 77 906–910 10.1128/AEM.01480-10 21131520 PMC3028700

[B8] Ferrari R. G. Galiana A. Cremades R. Rodrígues J. C. Magnani M. Tognin M. C. B. (2013a). Plasmid-mediated quinolone resistance (PMQR) and mutations in the topoisomerase genes of *Salmonella enterica* strains from Brazil. *Braz. J. Microbiol.* 44 657–662 10.1590/S1517-83822013000200046 24294265 PMC3833171

[B9] Ferrari R. G. Galiana A. Cremades R. Rodrígues J. C. Magnani M. Tognin M. C. B. (2013b). Expression of the marA, soxS, acrB and ramA genes related to the AcrAB/TolC eﬄux pump in *Salmonella enterica* strains with and without quinolone resistance-determining regions gyrA gene mutations. *Braz. J. Infect. Dis.* 17 125–130 10.1016/j.bjid.2012.09.011 23453941 PMC9427363

[B10] Gomes-Neto N. J. Luz I. S. Franco O. L. Magnani M. Souza E. L. (2014). Tolerance evaluation of *Salmonella enterica* serovar Typhimurium by exposure of subinhibitory amounts of *Rosmarinus officinalis* L. *essential* oil and 1,8-cineole in meat-model. *Int. J. Food Sci. Tech* 49 1912–1917 10.1111/ijfs.12522

[B11] Greenacre E. J. Brocklehurst T. F. (2006). The acetic tolerance response induces cross-protection to salt stress in *Salmonella* Typhimurium. *Int. J. Food. Microbiol.* 112 62–65 10.1016/j.ijfoodmicro.2006.05.012 16842874

[B12] Hammer K. A. Carson C. F. Riley T. V. (2012). Effects of melaleuca alternifolia (Tea Tree) essential oil and the major monoterpene component terpinen-4-ol on the development of single- and multistep antibiotic resistance and antimicrobial susceptibility. *Antimicrob. Agents Chemother.* 56 909–915 10.1128/AAC.05741-11 22083482 PMC3264233

[B13] Hendriksen R. S. Vieira A. R. Karlsmose S. Lo-Fo-Wong D. M. Jensen A. B. Wegener H. C. (2011). Global monitoring of Salmonella serovar distribution from the world health organization global foodborne infections network country data bank: results of quality assured laboratories from 2001 to 2007. *Foodborne Pathog. Dis.* 8 887–900 10.1089/fpd.2010.0787 21492021

[B14] Kottwitz L. B. M. Oliveira T. C. R. M. Alcocer I. Farah S. M. S. S. Abrahão W. S. M. Rodrigues D. P. (2010). Epidemiologic data of salmonellosis outbreaks occurred between 1999 and 2008 in in Paraná State, Brazil. *Acta Scientiarum.* 32 9–15.

[B15] Leyer G. J. Johnson E. A. (1993). Acid adaptation induces cross-protection against environmental stresses in *Salmonella* Typhimurium. *Appl. Environ. Microbiol.* 59 1842–1847. 8328803 10.1128/aem.59.6.1842-1847.1993 PMC182170

[B16] Luz I. S. Gomes-Neto N. J. Tavares A. G. Nunes P. C. Magnani M. Souza E. L. (2012). Evidence for lack of acquisition of tolerance in *Salmonella enterica* Serovar Typhimurium ATCC 14028 after exposure to sub-inhibitory amounts of *Origanum vulgare* L. essential oil and carvacrol. *Appl. Environ. Microbiol.* 78 5021–5024 10.1128/AEM.00605-12 22544235 PMC3416383

[B17] Luz I. S. Melo A. N. Bezerra T. K. Madruga M. Magnani M. Souza E. L. (2014). Subinhibitory amounts of *Origanum vulgare* L. essential oil and carvacrol cause injury and changes in membrane fatty acid of *Salmonella* Typhimurium cultivated in a meat broth. *Foodborne Pathog. Dis.* 11 357–361 10.1089/fpd.2013.1695 24588810

[B18] McMahon M. A. S. Blair I. S. Moore J. E. McDowell D. A. (2007). Habituation to sub-lethal concentrations of tea tree oil (*Melaleuca alternifolia*) is associated with reduced susceptibility to antibiotics in human pathogens. *J. Antimicrob. Chemother.* 59 125–127 10.1093/jac/dkl443 17071952

[B19] McMahon M. A. S. Tunney M. M. Moore J. E. Blair I. S. Gilpin D. F. (2008). Changes in antibiotic susceptibility in staphylococci habituated to sub-lethal concentrations of tea tree oil (*Melaleuca alternifolia*). *Lett. Appl. Microbiol.* 47 263–268 10.1111/j.1472-765X.2008.02420.x 18778374

[B20] Rushdy A. A. Mabrouk M. I. Abu-Sef F. A. Kheiralla Z. H. Mohamed A. S. Saleh N. M. (2013). Contribution of different mechanisms to the resistance to fluoroquinolones in clinical isolates of *Salmonella enterica*. *Braz. J. Infect. Dis.* 4 431–437 10.1016/j.bjid.2012.11.012 23742803 PMC9428056

[B21] Sarker S. D. Nahar L. Kumarasamy Y. (2007). Microtitre plate-based antibacterial assay incorporating resazurin as an indicator of cell growth, and its application in the in vitro antibacterial screening of phytochemicals. *Methods* 42 321–324 10.1016/j.ymeth.2007.01.006 PMC1895922 17560319

[B22] Shah J. Desai P. T. Chen D. Stevens J. R. Weimer B. C. (2013). Preadaptation to cold stress in *Salmonella enterica* serovar Typhimurium increases survival during subsequent acid stress exposure. *Appl. Environ. Microbiol.* 79 7281–7289 10.1128/AEM.02621-13 24056458 PMC3837722

[B23] Souza E. L. Azer êdo G. A. Sousa J. P. Figueiredo R. C. B. Q. Stamford T. L. (2013). Cytotoxic effects of *Origanum vulgare* L. and *Rosmarinus officinalis* L. essential oils alone and combined at subinhibitory amounts on *Pseudomonas fluorescens* in a vegetable broth. *J. Food Saf.* 33 163–171 10.1111/jfs.12036

[B24] Souza R. B. Magnani M. Ferrari R. G. Kottwitz L. B. M. Sartori D. Tognim M. C. B. (2011). Detection of quinolone-resistance mutations in *Salmonella* spp. strains of epidemic and poultry origin. *Braz. J. Microbiol.* 42 211–215 10.1590/S1517-83822011000100026 24031623 PMC3768919

[B25] Spector M. P. Kenyon W. J. (2012). Resistance and survival strategies of *Salmonella enterica* to environmental stresses. *Food Res. Int.* 45 455–481 10.1016/j.foodres.2011.06.056

[B26] Timme R. E. Allard M. W. Luo Y. Strain E. Pettengill J. Wang C. (2012). Draft genome sequences of 21 *Salmonella enterica* serovar Enteritidis strains. *J. Bacteriol.* 194 5994–5995 10.1128/JB.01289-12 23045502 PMC3486122

[B27] Yang Y. Khoo W. J. Zheng Q. Chung H. J. Yuk H. G. (2014). Growth temperature alters *Salmonella* Enteritidis heat/acid resistance, membrane lipid composition and stress/virulence related gene expression. *Int. J. Food Microbiol.* 172 102–109 10.1016/j.ijfoodmicro.2013.12.006 24368153

